# Genome-wide characterization of mitochondrial DNA methylation in human brain

**DOI:** 10.3389/fendo.2022.1059120

**Published:** 2023-01-16

**Authors:** Matthew Devall, Darren M. Soanes, Adam R. Smith, Emma L. Dempster, Rebecca G. Smith, Joe Burrage, Artemis Iatrou, Eilis Hannon, Claire Troakes, Karen Moore, Paul O’Neill, Safa Al-Sarraj, Leonard Schalkwyk, Jonathan Mill, Michael Weedon, Katie Lunnon

**Affiliations:** ^1^ Department of Clinical and Biomedical Sciences, Faculty of Health and Life Sciences, University of Exeter, Exeter, United Kingdom; ^2^ Institute of Psychiatry, Psychology and Neuroscience, King’s College London, London, United Kingdom; ^3^ School of Biological Sciences, University of Essex, Essex, United Kingdom

**Keywords:** 5-Methylcytosine (5mC), Brain, DNA Methylation, epigenetics, Mitochondria, mtDNA

## Abstract

**Background:**

There is growing interest in the role of DNA methylation in regulating the transcription of mitochondrial genes, particularly in brain disorders characterized by mitochondrial dysfunction. Here, we present a novel approach to interrogate the mitochondrial DNA methylome at single base resolution using targeted bisulfite sequencing. We applied this method to investigate mitochondrial DNA methylation patterns in post-mortem superior temporal gyrus and cerebellum brain tissue from seven human donors.

**Results:**

We show that mitochondrial DNA methylation patterns are relatively low but conserved, with peaks in DNA methylation at several sites, such as within the *D-LOOP* and the genes *MT-ND2*, *MT-ATP6*, *MT-ND4*, *MT-ND5* and *MT-ND6*, predominantly in a non-CpG context. The elevated DNA methylation we observe in the *D-LOOP* we validate using pyrosequencing. We identify loci that show differential DNA methylation patterns associated with age, sex and brain region. Finally, we replicate previously reported differentially methylated regions between brain regions from a methylated DNA immunoprecipitation sequencing study.

**Conclusions:**

We have annotated patterns of DNA methylation at single base resolution across the mitochondrial genome in human brain samples. Looking to the future this approach could be utilized to investigate the role of mitochondrial epigenetic mechanisms in disorders that display mitochondrial dysfunction.

## Introduction

Mitochondria are unique organelles in that they have their own genome, ~16.6kb in size, allowing for them to self-govern many processes such as replication. The human mitochondrial genome (mtDNA) consists of 37 genes, 22 encoding for transfer RNAs (tRNAs), two for ribosomal RNAs (rRNAs) and 13 that encode for proteins in the electron transport chain (ETC). The ETC itself consists of 97 genes (mitochondrial and nuclear) working together to orchestrate an effective response to cellular demands, in the form of oxidative phosphorylation. Thirteen of the ETC genes are mitochondrial-encoded; the remaining 84 are encoded by the nuclear genome and their protein products are imported into the mitochondrion (1). ETC proteins are directly involved in the regulation of cellular respiration, generating the majority of ATP required for the process. However, mitochondria play a vital role in a variety of key biological functions, including apoptosis, [as reviewed in (2)], the regulation of calcium homeostasis (3, 4) and the production of reactive oxygen species (ROS) (5).

Epigenetic processes mediate the reversible regulation of gene expression, occurring independently of DNA sequence variation and acting principally through chemical modifications to DNA and nucleosomal histone proteins. DNA methylation is the most stable and well-studied epigenetic mark. The advent of cost-effective epigenome-wide approaches, such as the Illumina Infinium^®^ 450K or EPIC BeadChip Arrays, have led to the identification of differentially methylated loci in a range of complex diseases, including for example Alzheimer’s disease (6), schizophrenia (7) cancer (8, 9) and type I diabetes (10). However, one caveat of this approach is the complete absence of coverage of the mitochondrial genome on these arrays. In 2011 there was a resurgence of interest in mitochondrial epigenetic studies, after both 5-methylcytosine (5mC) and 5-hydroxymethylytosine were reported in mtDNA. Furthermore, both DNA methyltransferase 1 (DNMT1) and S-adenosylmethionine (SAM) have been identified in the mitochondria (11, 12). As such, recent studies have suggested a role for differential mtDNA methylation in numerous pathologies characterized by mitochondrial dysfunction, including cancer (13), amyotrophic lateral sclerosis (14), and Alzheimer’s disease (15–18). However, until recently, studies have been limited to low-resolution immunohistochemical techniques (11) or candidate-based approaches such as methylation-specific pyrosequencing (19). To date, studies investigating DNA methylation across the mitochondrial genome have primarily utilized publicly available Methylated DNA immunoprecipitation sequencing (MeDIP-Seq) datasets (20, 21). However, these studies are limited to semi-quantitative calling of regional methylation and cannot quantify levels at specific cytosine bases. Furthermore, the presence of nuclear-mitochondrial pseudogenes (*NUMT*s) in the nuclear genome, which are regions of the mitochondrial genome that have translocated and inserted into the nuclear genome, leads to the requirement for the removal of homologous regions from the analysis (20, 21).

We have previously shown that a commercially available method from Miltenyi Biotech, which magnetically isolates mitochondria using anti-TOM22 antibodies, gives a significant enrichment of mtDNA to nuclear DNA (ncDNA) (22). Here we combine this method with a customized, targeted bisulfite sequencing approach to allow us to quantify mtDNA methylation patterns in post-mortem brain samples at single nucleotide resolution along the entire 16.569kb genome.

## Methods

### Sample demographics

Brain tissue was obtained from seven donors archived in the MRC London Neurodegenerative Disease Brain Bank (http://www.kcl.ac.uk/iop/depts/cn/research/MRC-London-Neurodegenerative-Diseases-Brain-Bank/MRC-London-Neurodegenerative-Diseases-Brain-Bank.aspx). From each donor, we sourced matched superior temporal gyrus (STG) and cerebellum (CER) brain tissue. The donors were selected with specific selection criteria in mind: similar age, distribution of sex across the group, low post-mortem intervals and no evidence of neurodegenerative disease. Sample demographics are shown in **Supplementary Table 1**.

### Mitochondrial isolation

Mitochondria were isolated from frozen, post-mortem brain tissue using a previously published method (22). This commercial method (Miltenyi Biotec) uses antibodies raised against the mitochondrial import receptor subunit TOM22, to enrich for mitochondria before DNA extraction using the QIAamp DNA Mini Kit (Qiagen). MtDNA was further enriched through the use of a custom library designed to amplify the mitochondrial genome (Agilent Technologies, California, USA).

### Custom capture of the mitochondrial epigenome

To capture the mitochondrial epigenome, a custom library of RNA baits (Agilent, California, U.S.A.: Design ID 0687721) was designed to provide 100% coverage of the genome at 5X tiling density. Isolated mtDNA extracted from frozen brain tissue was subjected to the Agilent 1μg Methyl-seq protocol. MtDNA was concentrated down to a total volume of 30μl DNA and 20μl (45ng/μl) of salmon sperm DNA (Sigma-Aldrich: 15632-011) was added to each sample as a carrier. Each sample was fragmented using the Bioruptor (Diagenode) for an extended period of 60 minutes. Samples were then processed according to the Agilent protocol with the following exceptions: a) Hybridization Buffer and Block Mix were made to specifications indicated on the protocol however, a 10% RNase block solution was made using 0.2µl RNase Block and 1.8µl of nuclease free water per reaction (instead of the 25% RNase block specified); b) given the size of the genome, only 2µl of the custom capture library was added to a PCR plate containing 2µl RNase block solution and 3µl of nuclease free water (instead of 5µl); c) the number of PCR cycles after bisulfite treatment was increased from 8 to 14 to adjust for low input levels. After generating indexed libraries for each sample, samples were then pooled in equimolar concentrations and sequenced using the Illumina HiSeq 2500 by Exeter Sequencing Service (Exeter, U.K.).

### Raw data processing

After sequencing, 100bp paired-end reads were de-multiplexed and following quality assessment using FastQC (23), reads were trimmed for adaptor content using TrimGalore (24). Using the same package, we also trimmed the eight base pairs from the 5’ and 3’ ends of both reads. Trimmed files were then aligned to the human reference genome GrCh38, through Bismark, using Bowtie 2 (25). Mapped reads were de-duplicated using the deduplicate_bismark function before CpG and non-CpG methylation was called by bismark_methylation_extractor. Only sites with read depth =>10 were considered for further analysis.The relationship between read depth and methylation level for sites with a read depth =>10 is shown in **Supplementary Figure 1**.

### Statistical analyses

All statistical analyses were carried out in R (v3.6.) (26). In our initial analysis we calculated mean percentage (%) DNA methylation for each site across the seven samples in each brain region separately. To investigate whether mtDNA methylation patterns were associated with specific covariates of interest (*e.g.* age, sex and brain region) we used a mixed effects model to control for the matched nature of our tissue samples. Briefly, this model used each of the covariates of interest (age, sex and brain region) as fixed effects and the individual as the random effect. To determine the significance of each covariate in isolation, a second (null) model was fitted, which didn’t contain the covariate of interest. An analysis of variance (ANOVA) was then performed between the two models to identify significantly differentially methylated bases for each covariate using lme4, a package within R (27). Nominal significance was deemed to be when P < 0.05 and to control for the effects of multiple testing, a Benjamani-Hochberg (BH) correction was applied to generate Q-values using the p.adjust function in R. To test for enrichment of trends found within our data, an exact binomial test was carried out using an in-built function within the R environment. To confirm DMRs between STR and CER tissues that we previously identified using MeDIP-seq (20), methylated cytosines were grouped into 100 bp non-overlapping regions and a paired t-test with Bonferroni correction was used to identify DMRs.

### Pyrosequencing

Bisulfite pyrosequencing was performed to confirm a peak in DNA methylation at the CpG site we identified in the *D-LOOP* (ChrM:545). Bisulfite conversion was performed using the Bisulfite-Gold kit (Zymo research, USA). A single amplicon (148bp) was generated using primers designed using the PyroMark Assay Design software 2.0 (Qiagen). DNA methylation was quantified using the Pyromark Q48 Autoprep system (Qiagen) following the manufacturer’s standard instructions and the PyroMark Q48 Autoprep 2.4.2 software. Bisulfite control regions showed a 97.5% conversion efficiency, with our 0% control showing methylation levels below the detection threshold.

## Results

### MtDNA methylation levels are relatively conserved across the genome

We used a customized, targeted bisulfite sequencing method to assess mtDNA methylation at single nucleotide resolution in isolated mtDNA from matched STG and CER brain tissue from seven donors free of neurodegenerative disease, including three males and four females. On average, 629,631 paired-end reads mapped to the mitochondrial reference genome per sample, providing an average sequencing depth of 6,384x (**Supplementary Table 2** **)**. On average, 57.77% of reads mapped to the mitochondrial genome, suggesting a large enrichment for mtDNA, exceeding levels we previously reported through the use of antibody-based enrichment alone (22).

In total, 7,174 methylated cytosines were analyzed for all samples (being covered by at least 10 sequence reads), of which 5,007 were present on the forward strand, due to a higher number of cytosines being present on this strand. The global average level of mtDNA methylation was low across all 7,174 sites (mean = 2.08%, standard deviation [SD] = 0.98%), corroborating previous assessments of global DNA methylation in the mitochondrial genome (20). This is in line with previous findings, suggesting that at many loci, mtDNA methylation levels are at non-biologically relevant levels across the genome (28). However, we were interested to see how much variability could be observed between mtDNA methylation at each methylated cytosine across the entire genome. To quantify this, the coefficient of variation (CV) - defined as the ratio of SD to the mean - was determined for each methylated site in STG and CER, respectively. Of the 7,174 methylated cytosines we assessed in the genome, 830 had a CV greater than one in the STG and 237 in the CER. Taken together, this shows that <2.75% of the assessed cytosines across both brain regions show inter-individual variation, suggesting a relatively conserved pattern of mtDNA methylation between individuals, although the number of variable sites is greater in STG samples.

### MtDNA methylation occurs with distinct prevalence at non-CpG sites

**Figure 1:**
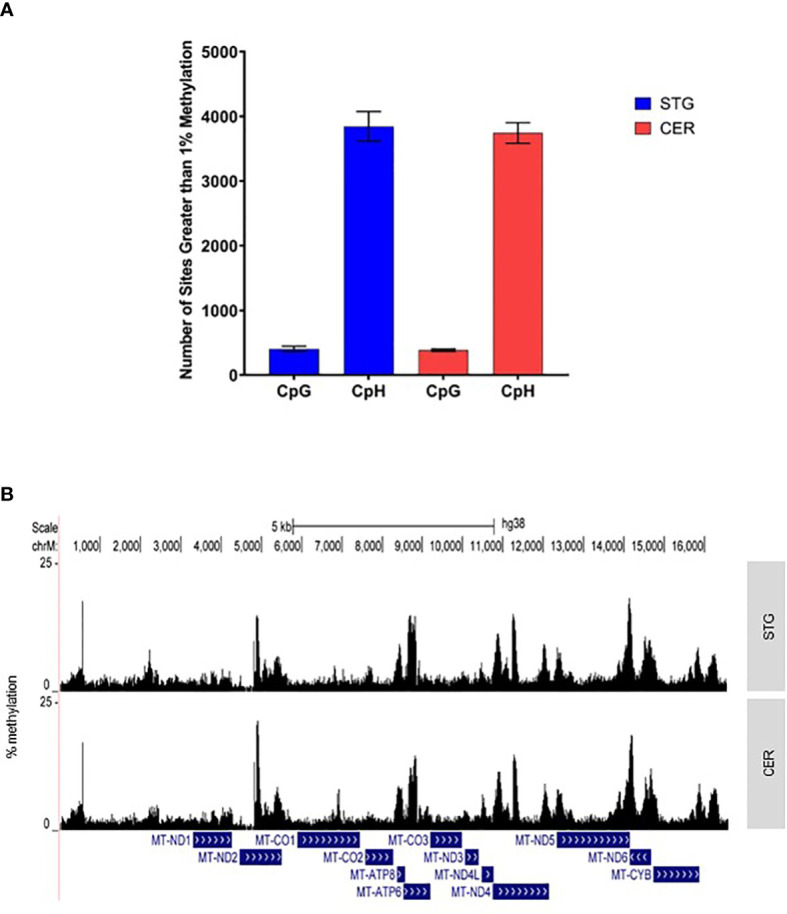
The mitochondrial genome is characterized by specific regions of methylation, predominantly in a CpH context. **(A)** Shown is the mean number of sites with >1% methylation in the STG (blue) and CER (red) in either a CpG or CpH context. Error bars represent the standard error of the mean (SEM). **(B)** Graph showing mean % methylation at each cytosine in the mitochondrial genome in STG (top panel) and CER (bottom panel) samples, created using UCSC browser (http://genome.ucsc.edu).

In mammals ncDNA methylation occurs predominantly in CpG contexts. However, DNA methylation has also been reported in non-CpG contexts (also termed CpH) in some mammalian cell types, including embryonic stem cells (ESCs), induced pluripotent stem cells (iPSCs) and human brain tissue (29). Although even within these cells, levels of CpH methylation are relatively low, for example in male, human embryonic stem cells, non-CpG methylation only comprises 25% of total methylated sites (30). However, previous studies have indicated a potential prevalence for methylation at non-CpG sites in mtDNA (31, 32). Of the 7,174 cytosines we captured in the current study, 6,319 (88%) were in a CpH context. Despite, the frequency of non-CpG methylation being higher than that previously reported in ncDNA, our data shows that the average level of non-CpG methylation in this study (~2.18%), is similar to average levels of non-CpG methylation within glial populations and about 2-3 fold lower than average neuronal methylation (33). However, whilst methylation levels were indeed low across the mitochondrial genome, on average, methylation was higher in a non-CpG context, with 60.85% (STG) and 59.24% (CER) of non-CpG sites displaying levels of methylation greater than 1%. In contrast, only 47.22% and 44.93% of methylation sites within a CpG context were at a level exceeding 1% in STG and CER, respectively (**Figure 1A**). Indeed, the overall average methylation level in a non-CpG context in STG (2.16%) and CER (2.20%) was much greater than that in a CpG context in either STG (1.39%) or CER (1.39%). Furthermore, of the 855 CpG sites captured, 19.4% and 2.92% of cytosines had a CV>1 in STG and CER, respectively, whilst of the 6,319 captured non-CpG sites, 10.5% (STG) and 3.35% (CER) had a CV>1. This suggests that levels of mtDNA methylation are far more variable between STG samples than CER samples.

### mtDNA methylation peaks are observed in the D-LOOP and MT-ND5

Whilst the average methylation level at each site was found to be low, a number of regions were identified with relatively high levels of mtDNA methylation (**Figure 1B** **;** **Table 1** **;** **Supplementary Table 3**). There were clear peaks of methylation within the genes *MT-ND2*, *MT-ATP6*, *MT-ND4*, *MT-ND5* and *MT-ND6*. The most highly methylated loci in the STG resided towards the end of the *MT-ND5* gene. We have previously shown, in our published MeDIP study, increasing levels of mtDNA methylation towards the end of this gene (13,800-14,100bp) (20). This is of particular interest as within the current study we observe relatively low levels of mtDNA methylation between 12,337bp-13,700bp, which encompasses the bulk of this gene, with only 26.6% (STG) and 25.7% (CER) of all sites in that region exhibiting levels of methylation greater than 2%. In contrast, between 13,801-14,144bp, 77.6% and 82.6% of all sites exceeded this 2% methylation level in the STG and CER, respectively, suggesting that a conserved pattern of mtDNA methylation is present in this genomic region across brain regions, which can be observed with different sequencing technologies. The ten most highly methylated loci in the CER were all located in the *MT-ND2* gene. However, this region was not analyzed in our previous MeDIP study due to homology with known *NUMTs*. As expected, the majority of the most highly methylated loci were in a CpH context: only four of the 100 most methylated sites were at a CpG (**Supplementary Table 3**). The most highly methylated CpG we identified in the STG was at 545bp (mean = 16.23%, SD = 6.91%), which also showed high levels of mtDNA methylation in the CER (mean = 15.72%, SD = 2.45%). This site lies within the mitochondrial displacement loop (*D-LOOP*), the major regulatory region of the mitochondrial genome and we previously identified a relatively high peak in the mitochondrial *D-LOOP*, including the 500-600bp window in our MeDIP study (20).

Table 1The 10 most highly methylated sites in mtDNA.

**A:** 

Genomic position	Context	Strand	Gene	Methylation% STG	Methylation% CER
14142	CpH	+	MT-ND5	16.85 (6.39)	15.38 (9.21)
545	CpG	–	D-LOOP	16.23 (6.91)	15.72 (2.45)
14136	CpH	+	MT-ND5	15.91 (6.55)	14.27 (8.13)
14130	CpH	+	MT-ND5	15.78 (6.30)	14.38 (7.09)
14131	CpH	+	MT-ND5	15.40 (6.37)	13.97 (7.70)
14129	CpH	+	MT-ND5	15.31 (6.40)	14.29 (7.52)
14137	CpH	+	MT-ND5	15.19 (6.47)	14.35 (8.64)
14144	CpH	+	MT-ND5	15.05 (6.46)	15.61 (9.11)
14169	CpH	+	MT-ND6	14.94 (6.27)	17.08 (9.30)
14125	CpH	+	MT-ND5	14.90 (6.11)	13.72 (7.13)

**B:** 

Genomic position	Context	Strand	Gene	Methylation% CER	Methylation% STG
4882	CpH	+	MT-ND2	19.61 (5.63)	13.48 (4.41)
4881	CpH	+	MT-ND2	19.01 (4.97)	12.78 (4.13)
4862	CpH	+	MT-ND2	18.97 (3.12)	12.27 (6.10)
4880	CpH	+	MT-ND2	18.75 (4.72)	12.75 (4.16)
4875	CpH	+	MT-ND2	18.65 (4.36)	13.42 (3.95)
4886	CpH	+	MT-ND2	18.60 (5.94)	13.17 (3.80)
4888	CpH	+	MT-ND2	18.44 (4.91)	13.42 (4.18)
4869	CpH	+	MT-ND2	18.34 (3.27)	12.77 (6.65)
4879	CpH	+	MT-ND2	18.28 (4.45)	13.01 (6.96)
4892	CpH	+	MT-ND2	18.14 (5.46)	13.68 (4.45)

Shown are genomic position (bp), strand, gene and average % mtDNA methylation level (± standard deviation) for the 10 most highly methylated loci in the STG (A) and CER (B).

**Figure 2:**
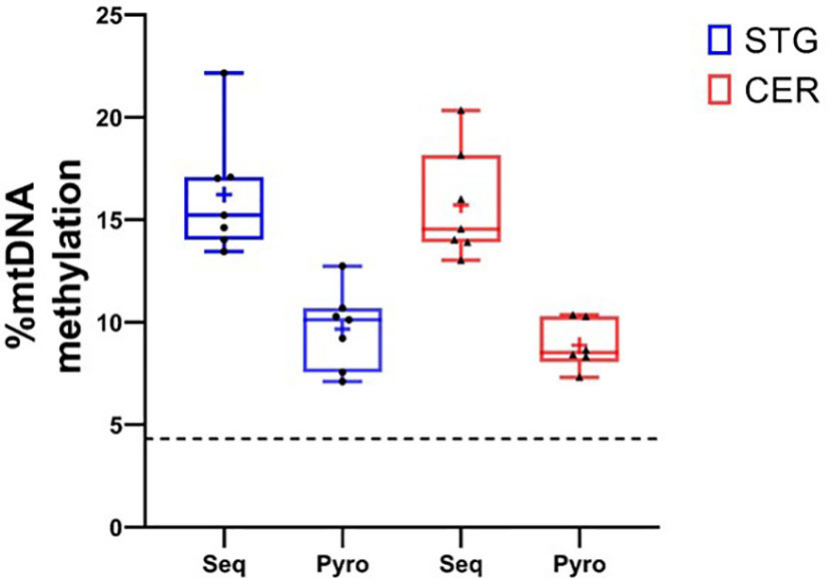
Validation of mtDNA methylation in the D-Loop using pyrosequencing. Box plots showing % methylation levels at site ChrM:545 in the seven donors used in the study, when profiled using targeted bisulfite sequencing (Seq) and pyrosequencing (Pyro) in the STG (blue) and CER (red). Boxplots represent the median (central line) and interquartile range (perimeter), with plus sign denoting the mean, whiskers showing the minimum and maximum value, and individual datapoints shown as black circles (STG) or triangles (CER). The dashed black horizontal line represents the reported minimum threshold for detecting methylation using pyrosequencing (4.3%).

In order to confirm our finding of a peak in DNA methylation in the mitochondrial D-LOOP we used pyrosequencing to measure mtDNA methylation at the CpG site at position 545bp in the same samples. Using this approach, we did observe detectable DNA methylation at this locus in both the STG (mean = 9.68%, SD = 1.78) and CER (mean = 9.38%, SD = 1.47) (**Figure 2**). Although the levels measured using pyrosequencing were lower than the targeted mitochondrial sequencing approach, these were significantly higher (P = 8.52 x 10^-10^) than the mean methylation level for all mitochondrial CpG sites measured in our targeted sequencing data (STG: mean = 1.39%, SD = 1.59; CER: mean = 1.39%, SD = 1.73), and were above the reported minimum threshold for detecting methylation using pyrosequencing (4.3%) (34).

### MtDNA methylation exhibits sex specific patterns across the genome

Given that ncDNA methylation patterns are strongly associated with sex at specific sites in the genome (35), we were interested to see whether the mtDNA methylome exhibited sex-specific patterns. Our initial analysis used a mixed effects model, controlling for the matched nature of our tissue samples allowing us to assess the effects of sex and other co-variates of interest (*i.e.* age, brain region). We identified 1,311 nominally significant (P < 0.05) differentially methylated positions (DMPs) associated with sex (**Supplementary Table 4**), of which 1,249 were hypomethylated in females. Using an exact binomial test, we found that this represented a significant enrichment for hypomethylation in females (P < 2.2 x 10^-16^). Of the 1,311 sites we identified, 19 loci remained after multiple testing correction using the Benjamani-Hochberg (BH) method (Q < 0.1) (**Supplementary Figure 2**). Of interest, 15 of these 19 sites were found in a non-CpG context. All of the CpH sites with Q *<* 0.1 were hypomethylated in females, whilst one of the BH-significant CpG sites was hypermethylated in females.

### MtDNA methylation tends to increase with age at significant DMPs across the genome

Alterations in mitochondrial function have been associated with age, as reviewed by Amigo et al. (36). We were interested to investigate whether age may alter mtDNA methylation patterns. Our mixed effects model identified 1,312 sites nominally significantly associated DMPs with age (**Supplementary Table 5**, **Supplementary Figure 3**), of which 1,246 were positively correlated with age, which represented an enrichment for hypermethylation in age-associated DMPs (P < 2.2 x 10^-16^). Of the 1,312 age-associated loci, 259 remained after multiple test corrections (Q < 0.1), of which 257 were positively correlated with increasing age.

### MtDNA methylation varies significantly between anatomical regions of the brain

In previous work, we have identified tissue-specific differentially methylated regions (DMRs) in mtDNA across different brain regions using MeDIP-Seq (20). As such, we were interested in investigating whether differences in mtDNA could be identified at single cytosine resolution between anatomically distinct brain regions. In our mixed effects model, we identified 759 nominally significant (P < 0.05) differences in mtDNA methylation across the genome that were associated with different brain regions (**Supplementary Table 6**). We identified 13 loci that remained significant after multiple testing corrections (Q < 0.1), of which seven were hypermethylated in the STG. Further, of the 759 sites, 435 were found to be hypermethylated in STG relative to CER; a binomial test showed that this represented a significant enrichment of hypermethylation in the STG (P = 6.35 x 10^-5^). To determine whether regional methylation patterns observed in our previous work were reproduced here, 100bp windows were generated across the mitochondrial genome by averaging methylation of all sites across each window in our current data. The average profile for each brain region across the mitochondrial genome was then plotted **(** **Supplementary Figure 4** **)**. Given the small sample size of both cohorts, varying distribution of epidemiological factors and the lack of data retained in the first study, it is unsurprising that few significant hits can be replicated. However, of the significant DMRs previously identified (20), four were also significant (t-test Q < 0.05) and showed the same direction of effect, including hypomethylation in the STG at one DMR (4001-4100bp: *MT-ND1*: Q = 1.8 x 10^-7^), and hypermethylation in the STG at three DMRs (12701-12800bp: *MT-ND5*, Q = 0.0032; 14501-14600bp: *MT-ND6*, Q = 0.0025; 15401-15500bp: *MT-CYB;* Q = 0.0020) **(** **Supplementary Figure 5** **)**.

## Discussion

Here, we present the first genome-wide map of mtDNA methylation at single nucleotide resolution in human brain tissue. This data resource can be accessed and explored *via* our website (www.epigenomicslab.com/mitochondria-dna-methylation-map/). Previous studies have investigated changes across the mitochondrial epigenome using publicly available data (21, 28). However, these studies have been limited by low sequencing depth, the use of non-isolated mtDNA, and in the case of MeDIP-Seq, relatively low resolution. Hong et al., used publicly available BS-Seq data to show that mtDNA methylation does not exist at biologically relevant levels (<1%). However, an ability to determine this conclusively, given the multi-copy nature of the mitochondrial genome, as well as the relatively low sequencing depth derived from non-enriched BS-Seq, may have led to an underrepresentation of true mtDNA methylation levels.

In our study, although average levels of mtDNA methylation across the 7,174 sites interrogated were low, there was considerable variability in methylation across the genome. Interestingly, a CpG site of the *D-LOOP*, 545bp, was found to have relatively high levels of mtDNA methylation across all samples and was validated using pyrosequencing. This adds further evidence to a study that identified high levels of mtDNA methylation across the mitochondrial *D-LOOP* (31), potentially highlighting a region of the mitochondrial genome which could have an important impact on the organelle’s function. Importantly, whilst a recent study suggested that relatively high levels of mtDNA methylation can be found in the *D-LOOP*, due to the triple helix nature of the region leading to incomplete bisulfite conversion (37) and over-estimation of methylation. To address this caveat in our study we have used an extended sonication period to further linearize mtDNA to reduce the effects of the secondary structure around this region. Furthermore, our study also finds consistent high levels of mtDNA methylation in other regions, for example *MT-ND2, MT-ATP6, MT-ND4, MT-ND5* and *MT-ND6*, which are not affected by this secondary structure.

Establishing the extent to which confounders such as age, sex and brain region play a role in ncDNA methylation studies has led to a more robust and reliable approach to epigenetic studies. However, previous studies into mtDNA methylation have not studied the genome-wide effect of these factors. Our preliminary analysis identified many nominally significant DMPs for all of the covariates, with 13 brain region, 259 age and 19 sex associated DMPs remaining significant after BH corrections for multiple testing. The small sample size of our study, as well as the lack of functional work carried out on these DMPs makes it difficult to ascertain the physiological relevance of the differences. However, the identification of a DMP in the *D-LOOP*, a region known to regulate mitochondrial transcription and replication, could possibly effectuate changes in overall mitochondrial output. Additionally, the finding that there is an enrichment for hypomethylated DMPs in females and that there is an enrichment for hypermethylated DMPs associated with increasing age highlights the need for carefully controlled, future epidemiological studies.

Age-related changes have also been identified in other mtDNA methylation studies (38, 39). Hypomethylation in ncDNA has been frequently attributed to increasing age (40) and age-associated hypermethylation has therefore been suggested to play a role in age–related disorders (41). As such, it is of interest to investigate why mtDNA methylation tends to increase across the genome with age. If it is to be assumed that mtDNA methylation exists at a relatively low level across the mitochondrial genome, then increases in mtDNA methylation could be a sign of dysregulation within the mitochondria. It is therefore possible that this general increase across the genome could be attributed to the increase in mitochondrial dysfunction seen during aging (42, 43), potentially by altering regulation of mtDNA expression. Despite this, few functional studies have investigated the effect of changes in mtDNA methylation, although mtDNA hypermethylation was shown to be associated with apoptosis in diabetic retinopathy (44). However, to date, no study has looked at the effect of multiple, site-specific mtDNA methylation changes occurring simultaneously.

The ubiquitous nature of mitochondrial distribution throughout the body, except for in red blood cells, highlights the importance of maintaining correct mitochondrial function. Despite levels of mtDNA methylation being relatively low in comparison to ncDNA methylation, a recent study identified that those individuals with increased mtDNA methylation levels at a site within *MT-RNR1* were associated with increased mortality risk when followed up nine years later (38). Studies have shown that mtDNA methylation changes at specific sites in the *D-LOOP* negatively correlate with changes in gene expression. One study demonstrated that increases in *D-LOOP* methylation were associated with reduced *MT-CYB*, *MT-ND6*, and *MT-COXII* transcript levels (44), whilst another showed that reduced *D-LOOP* methylation was associated with increased *MT-ND2* expression in colorectal cancer tumour tissue (45). One recent study has demonstrated decreased mtDNA methylation in the *D-LOOP* in blood DNA in Alzheimer’s disease patients compared to non-demented controls (18). Previous studies have demonstrated that mtDNA methylation is typically very low, with levels of approximately 0.5 – 1.0% (33). However, we have identified several cytosines with DNA methylation levels greater than 5% across the mitochondrial genome. However, we were unable to identify a clear correlation between increased frequency of methylation at these sites and any of the covariates tested in our analyses. As such, clues to their biological importance remain to be elucidated.

Despite our efforts, there are several key limitations of this study. Most importantly, whilst age-related hypermethylation changes were observed in this cohort, given the small sample size, most ages were only represented once and overall, the age range of samples used in the study was relatively small. It is therefore possible that the effects of age on DNA methylation merely represent inter-individual differences and further studies including a broader range of ages, with larger numbers of both males and females should be carried out in the future. It is worth noting that the effect sizes we observed with respect to age and sex, were relatively small, and further studies in larger cohorts will elucidate whether these are representative of a larger population and whether these are likely to be biologically meaningful. A recent study has highlighted that mtDNA is extensively methylated during embryogenesis to prevent oxidative-stress-induced mtDNA damage (46) and therefore it would be of interest to measure mtDNA methylation across the human lifespan. Another limitation of our study is that given the small sample size we have been unable to explore how mtDNA genetic differences may influence mtDNA methylation. Interestingly, a recent study by Laaksonen and colleagues has identified mtDNA quantitative trait loci (QTLs) whereby mtDNA genetic variation controls DNA methylation at specific sites in the nuclear genome (47). However, to the best of our knowledge, no study has yet explored how mtDNA genetic variation impacts on mtDNA methylation, which warrants investigation in the future.

Our study has used bisulfite-treated DNA, and there is a small possibility that the very low methylation levels could be due to inefficient bisulfite conversion, although we have attempted to mitigate this by using an extended sonication period and by comparing some of our results to MeDIP-seq data. Another technology that overcomes this limitation is long-read sequencing technologies, such as the Oxford Nanopore Technologies (ONT) platform, which can detect DNA methylation on native DNA based on changes in electric current as a DNA molecule moves through the nanopore. A recent study by Bicci and colleagues used this technique to quantify mtDNA methylation at single nucleotide resolution in different cell lines and human tissues (48). The authors reported that methylation at CpG sites was below the background threshold. However, the calling algorithm they utilized did not assess CpH sites and they did not analyze human brain tissue. Given that we report that mtDNA methylation is higher and more prevalent at non-CpG sites in human brain, it would be of interest in the future to use this technology, in conjunction with algorithms that call CpH methylation, for example Megaladon (49), Tombo (50) and DeepSignal (51), to study mtDNA methylation in human brain. In addition, as ncDNA methylation at CpH sites is enriched in the brain compared to other tissues (29), it would be of interest in the future to profile mtDNA methylation using these technologies in other tissues, to determine whether mitochondrial CpH methylation is enriched in the brain, or is present across all human tissues.

## Conclusions

We present the first, genome-wide analysis of mtDNA methylation in human brain tissue at single base resolution, providing a map of mtDNA methylation across human brain as well as identifying interesting differences in mtDNA methylation between different brain regions, sexes and with age. This not only highlights a potential biological importance for mtDNA methylation but also highlights the need to control for these factors in future studies. Our study provides the building blocks for further research into the role of mtDNA methylation in the brain, particularly in diseases characterized by mitochondrial dysfunction.

## Data availability statement

The datasets presented in this study can be found in online repositories. The names of the repository/repositories and accession number(s) can be found below: NCBI Gene Expression Omnibus (GEO): Dataset GSE189565.

## Ethics statement

The studies involving human participants were reviewed and approved by NHS South East London REC 3. Written informed consent was not provided because Post-mortem tissue used.

## Author contributions

MD, AS, ED, JB, AI and KM conducted laboratory experiments. MD, DS, RS, EH, PO, LS and MW undertook data analysis and bioinformatics. CT, SA-S and JM provided samples for analysis. KL and MW conceived and supervised the project. MD, DS and KL drafted the manuscript. All authors contributed to the article and approved the submitted version.
