# Supplementary material for: Genome-wide characterization of mitochondrial DNA methylation in human brain
Source: Front Endocrinol (Lausanne). 2023 Jan 16;13:1059120. doi: 10.3389/fendo.2022.1059120 (PMC9885148; doi:10.3389/fendo.2022.1059120)
Supplement: Supplementary file 1 [file DataSheet_1.docx]

**SUPPLEMENTARY FIGURES**

**Supplementary Figure 1: A Scattergraph showing the relationship between (log10) mean sequence coverage and mean % methylation for all the methylation sites used in this study.** Only sites with a read depth =>10 were utilized in our analyses.

**
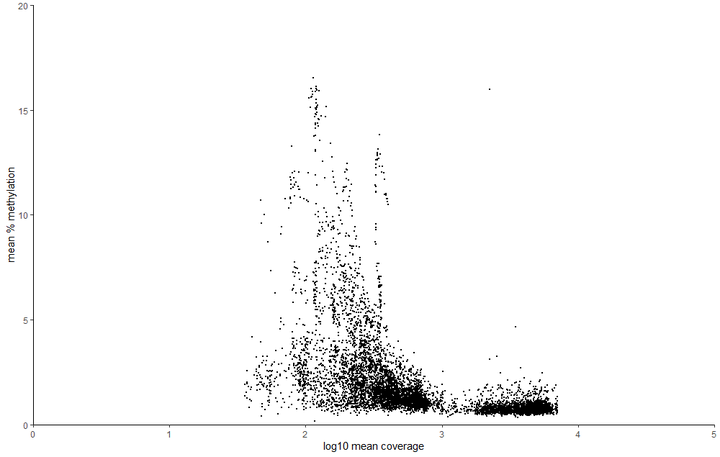
**

**Supplementary Figure 2: Sex-specific patterns of mtDNA methylation are seen in human brain tissue.** A boxplot showing mtDNA methylation patterns in females (orange) and males (green) at 19 loci that showed significant sex differences after multiple testing (Q < 0.1) in our mixed effects model, with 18 of these loci show hypomethylation in females. Boxplots represent the median (central line) and interquartile range (perimeter), with plus sign denoting the mean and whiskers showing the minimum and maximum value.


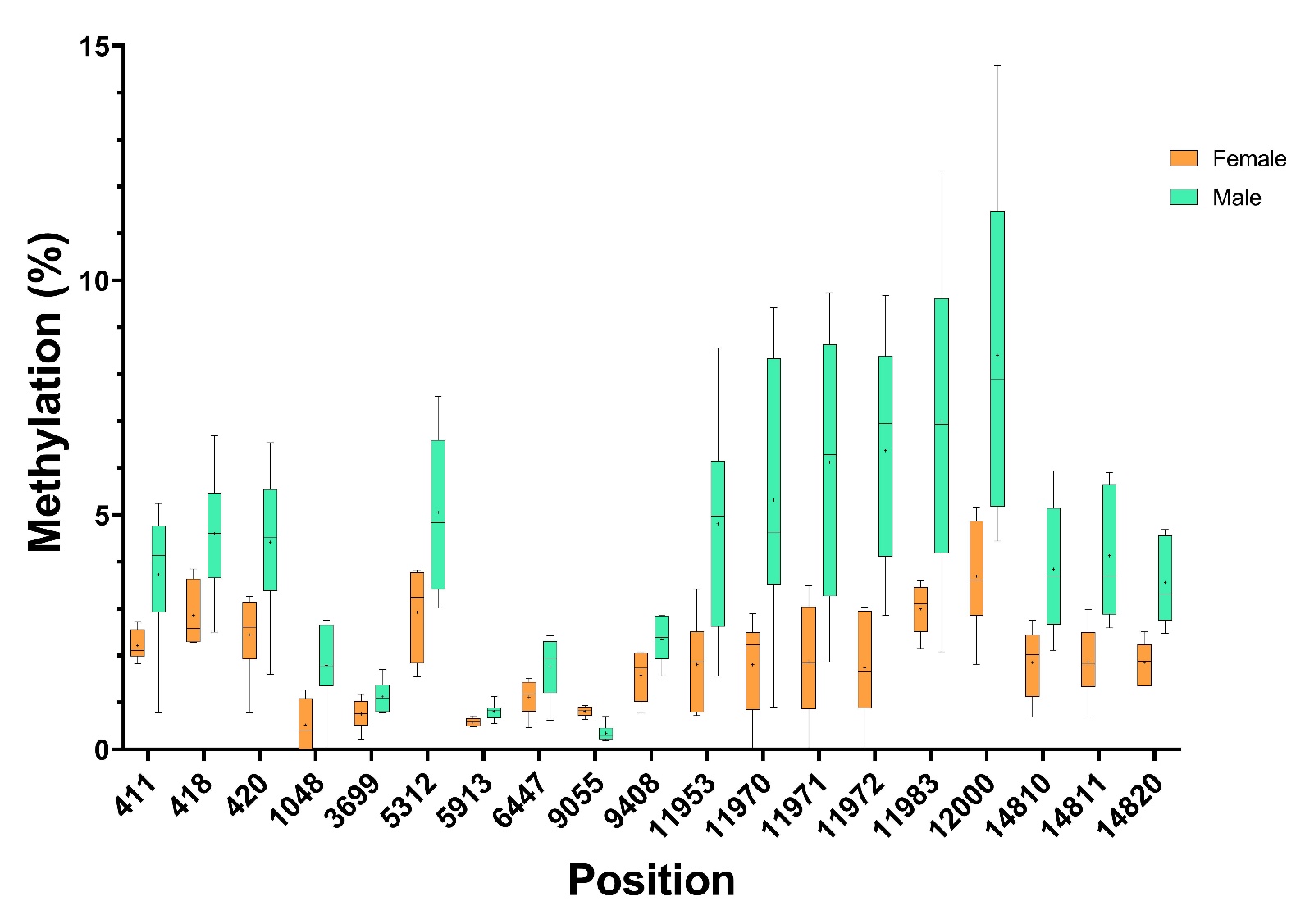


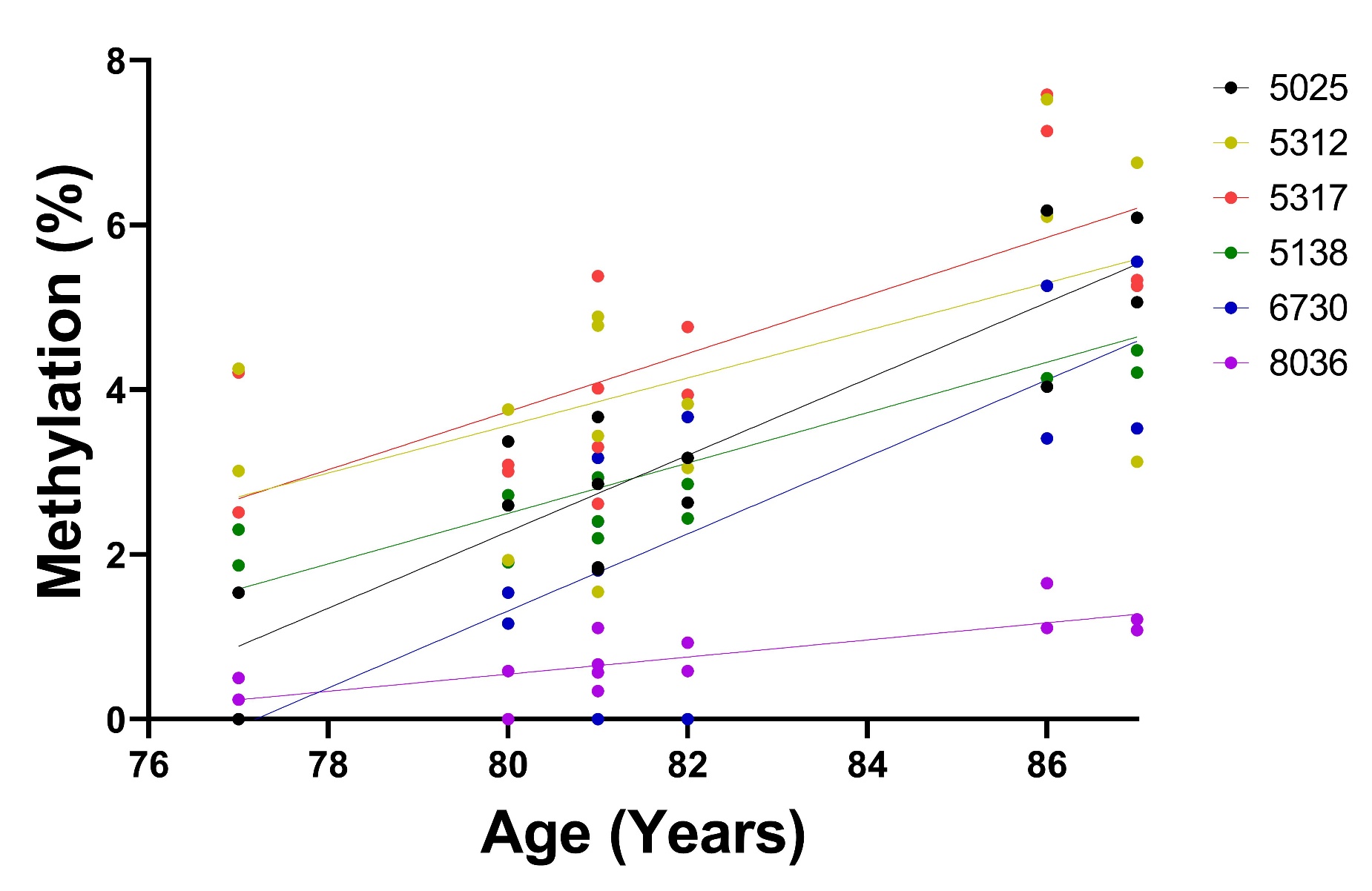
**Supplementary Figure 3: The top six loci that were most significantly differentially methylated with age in our mixed effects model.** Plotted is the % methylation in each sample for six positions: 5025bp (black), 5138bp (green), 5312 bp (yellow), 5317 bp (red) (all in *MT-ND2*), 6730 bp (*MT-COX1*: blue), 8036 bp (*MT-COX2*: purple). Regression lines have been plotted for each of these six loci (in the corresponding color) to highlight that methylation increases with age.

**Supplementary Figure 4:** **DNA methylation patterns in 100bp windows across the mitochondrial genome.** Shown are mean mtDNA methylation levels per 100bp window in the STG (blue) and CER (red).


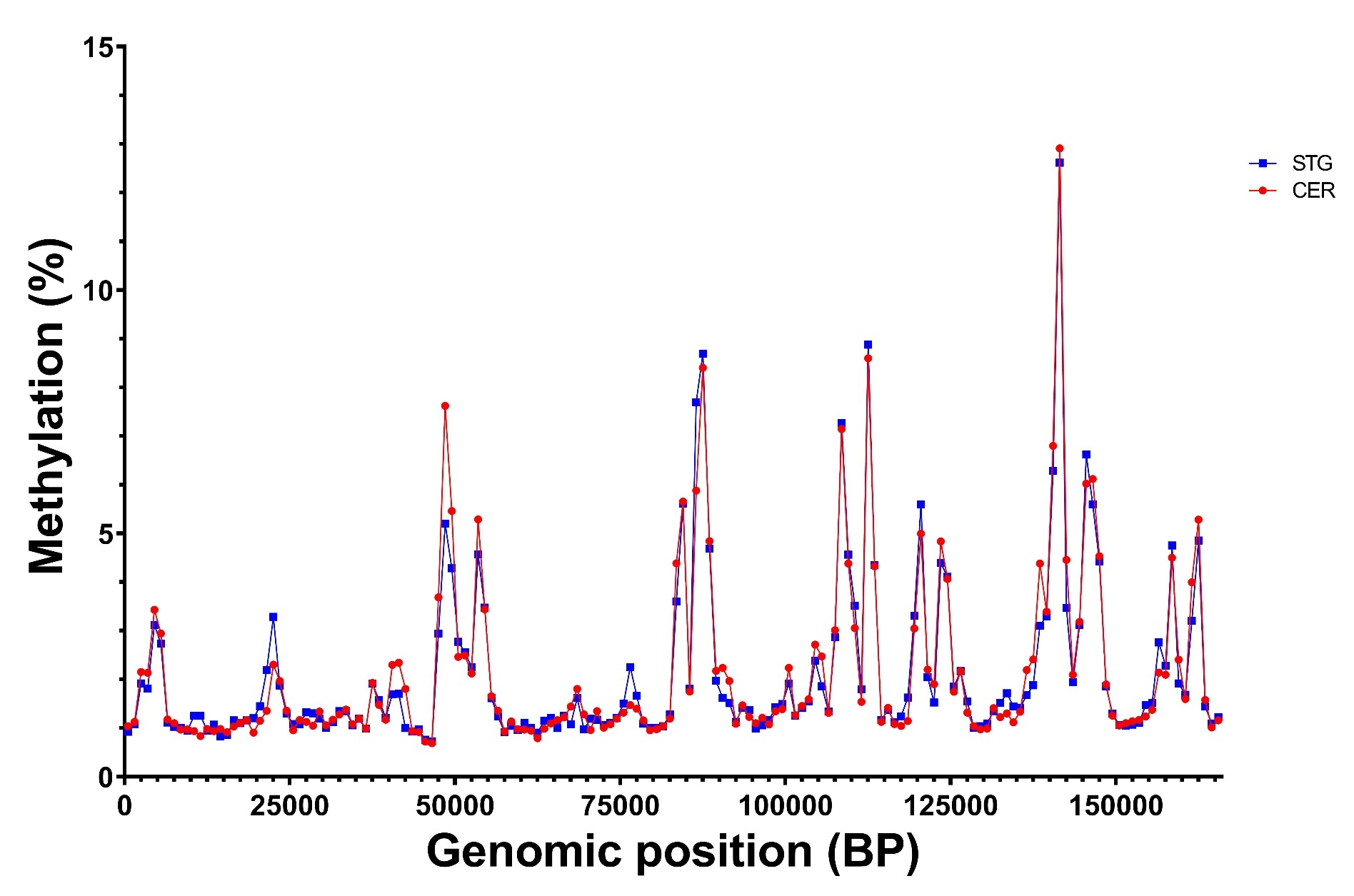


**Supplementary Figure 5: Replication of previously identified tissue DMRs.** In the current study we looked in 100bp windows across the mitochondrial genome and compared these to tissue-type DMRs we had previously identified using MeDIP-seq. Four of our previous tissue specific DMRs were replicated, residing in *MT-ND1* (4001-4100bp, Q = 1.82 x 10^-7^), *MT-ND5* (12701- 12800bp, Q = 0.00318), *MT-ND6* (14501-14600bp, Q= 0.00251) and *MT-CYB* (15401-15500bp, Q = 0.00197). Samples from the STG were hypermethylated in three of the DMRs, confirming the previous results found using MeDIP. Shown are the mean % methylation levels (+/- SEM) in the 100bp windows in STG (blue) and CER (red).


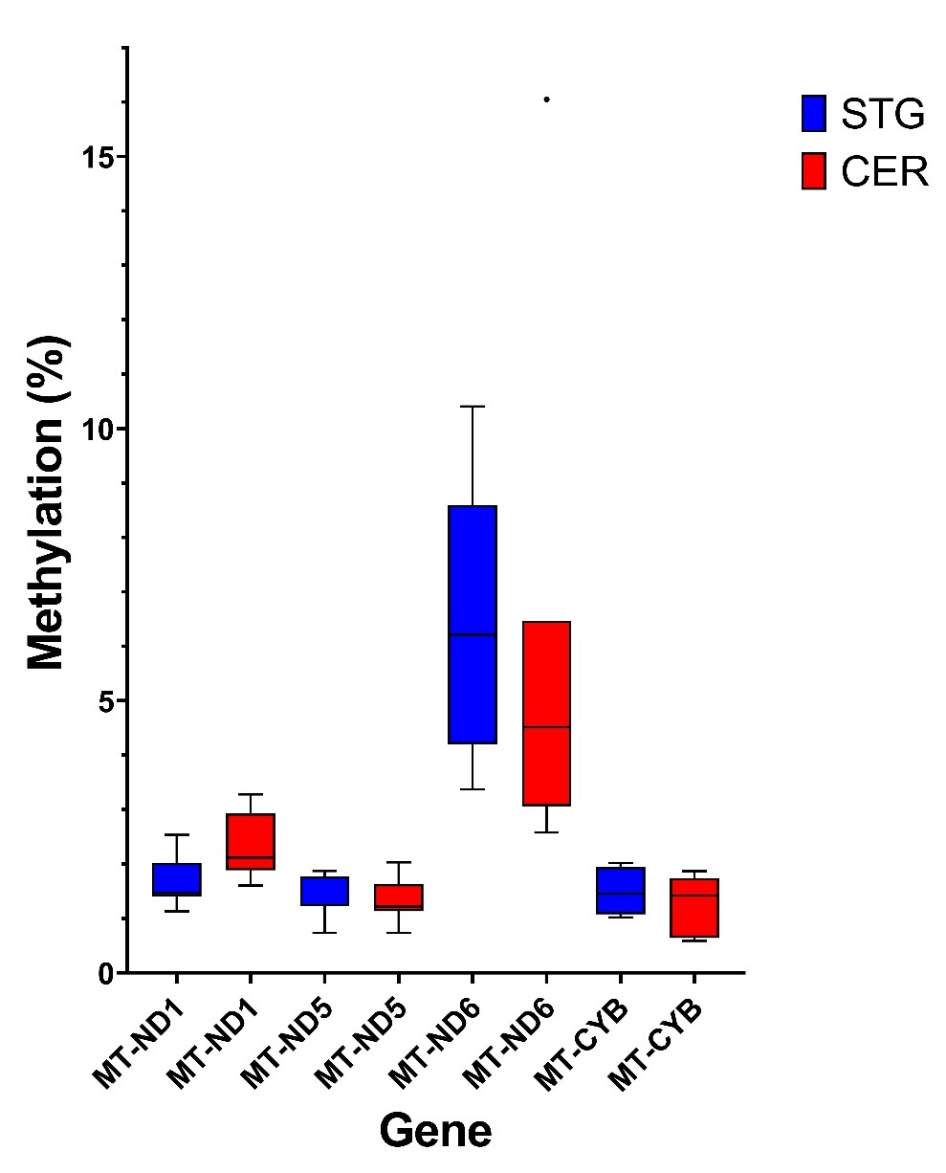


**MT-ND1 MT-ND5 MT-ND6 MT-CYB**
